# Recombinant pharmaceuticals from microbial cells: a 2015 update

**DOI:** 10.1186/s12934-016-0437-3

**Published:** 2016-02-09

**Authors:** Laura Sanchez-Garcia, Lucas Martín, Ramon Mangues, Neus Ferrer-Miralles, Esther Vázquez, Antonio Villaverde

**Affiliations:** Institut de Biotecnologia i de Biomedicina, Universitat Autònoma de Barcelona, 08193 Bellaterra, Cerdanyola del Vallès, Spain; Departament de Genètica i de Microbiologia, Universitat Autònoma de Barcelona, 08193 Bellaterra, Cerdanyola del Vallès, Spain; CIBER de Bioingeniería, Biomateriales y Nanomedicina (CIBER-BBN), 08193 Bellaterra, Cerdanyola del Vallès, Spain; Technology Transfer Office, Edifici Eureka, Universitat Autònoma de Barcelona, 08193 Bellaterra, Cerdanyola del Vallès, Spain; Institut d’Investigacions Biomèdiques Sant Pau, Josep Carreras Research Institute and CIBER-BBN, Hospital de la Santa Creu i Sant Pau, Barcelona, Spain

**Keywords:** Recombinant proteins, Protein drugs, Recombinant DNA, Fusion proteins, Biopharmaceuticals

## Abstract

Diabetes, growth or clotting disorders are among the spectrum of human diseases related to protein absence or malfunction. Since these pathologies cannot be yet regularly treated by gene therapy, the administration of functional proteins produced ex vivo is required. As both protein extraction from natural producers and chemical synthesis undergo inherent constraints that limit regular large-scale production, recombinant DNA technologies have rapidly become a choice for therapeutic protein production. The spectrum of organisms exploited as recombinant cell factories has expanded from the early predominating *Escherichia coli* to alternative bacteria, yeasts, insect cells and especially mammalian cells, which benefit from metabolic and protein processing pathways similar to those in human cells. Up to date, around 650 protein drugs have been worldwide approved, among which about 400 are obtained by recombinant technologies. Other 1300 recombinant pharmaceuticals are under development, with a clear tendency towards engineered versions with improved performance and new functionalities regarding the conventional, plain protein species. This trend is exemplified by the examination of the contemporary protein-based drugs developed for cancer treatment.

## Background

Human cells produce thousands of proteins that integrated into an extremely complex physiologic network perform precise actions as catalysers, signalling agents or structural components. Then, dysfunction of proteins with abnormal amino acid sequences or the absence of a given protein often results in the development of severe pathologies such as diabetes [1], dwarfism [2], cystic fibrosis [3], thalassaemia [4] or impaired blood clotting [5], among many others [6, 7]. In the absence of standardized gene therapy treatments that would genetically reconstitute functional protein production within the patient, protein deficiencies must be treated by the punctual or repeated clinical administration of the missing protein, so as to reach ordinary functional concentrations. These therapeutic proteins are produced ex vivo mostly in biological systems [8], which must guarantee not only full protein functionalities but also a cost-effective industrial fabrication and the absence of hazardous contaminants. Protein drugs have to necessarily conform to quality constrains stricter than those expected in the production of enzymes for chemical industries, which consequently defines the choice of recombinant hosts, protocols and production strategies. Nowadays, there are over 400 marketed recombinant products (peptides and proteins) and other 1300 are undergoing clinical trials (figures updated on May 2015 [9]).

In this context of expanding protein drug markets, there is a generic consensus about the need to enable drugs for cell- or tissue-targeted delivery to reduce doses, production costs and side effects. While increasing protein stability in vivo can be reached by discrete modifications in the amino acid sequence, generating fusions between therapeutic proteins and specific peptide ligands or antibodies that interact with particular cell receptors might allow acquiring specificity in the delivery process. In this regard and also pushed by the convenience to combine diagnosis and therapy in theranostic agents [10, 11], contemporary research on protein pharmaceuticals tends towards engineered versions functionally more sophisticated than plain natural polypeptides.

## Review

### Cell factories

**Fig. 1:**
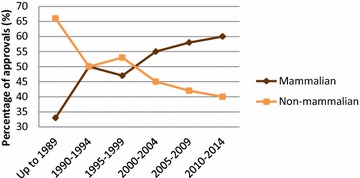
Number of recombinant protein products approved for use as drugs in humans, depending on the type of production platform

Since early recombinant DNA times, ever-increasing understanding of cell physiology and stress, and of factors involved in heterologous gene expression and protein production empowered the use of different living factories, namely prokaryotic and eukaryotic cells, plants or animals [12, 13]. By using these systems, recombinant production solves source availability problems, is considered a bio-safe and green process and confers the ability to modify amino acid sequences and therefore protein function, to better adjust the product to a desired function [14]. There is a wide and growing spectrum of expression systems that are becoming available for the production of recombinant proteins [15, 16]. *Escherichia coli* was the prevalent platform when the biopharmaceutical sector emerged in the 1980s, and it was followed by the implementation of the yeast *Saccharomyces cerevisiae*. Both systems and the associated genetic methodologies exhibit an unusually high versatility, making them adaptable to different production demands [17]. Despite the exploration of insect cells as initially successful system especially for vaccine-oriented proteins, mammalian cell lines (most notably CHO cells) are nowadays the prevailing animal-derived cell system due to their suitability to produce conveniently glycosylated proteins [18, 19] (Fig. 1). The ability to carry out post translational modifications contrasts with complex nutritional requirements, slow growth and fragility, and relatively high production timing and costs. Thus, among many conventional and emerging cell-based systems for protein production, bacteria, yeast and mammalian cell lines are the most common in biopharma, and both prokaryotic and eukaryotic systems are constantly evolving and competing to improve their properties and intensify as platforms of choice for protein drug production [14]. While bacteria has lost its early leading role in the field [19], about 30 % of marketed biopharmaceuticals are still produced in this system [20], as supported by the unusual physiological and genetic manipulability of prokaryotic cells [21].

In fact, the main purpose in the development of new protein production platforms is to enhance drug functionality through reaching successful protein folding and post-translational modifications, while keeping the low complexity and high flexibility associated to prokaryotic cell culture. In this context, Gram-positive bacteria such as *Bacillus megaterium* [22] and *Lactococcus lactis* [23] allow efficient protein secretion in absence of endotoxic cell wall components, while filamentous fungi (such as *Trichoderma reesei*, [24]), moss (*Physcomitrella patens*, [25, 26]) and protozoa (*Leishmania tarentolae*, [27–29]) promote glycosylation patterns similar to those in mammalian proteins but being still cultured through methods simpler than those required by mammalian cells. Extensive descriptions of emerging (bacterial and non-bacterial) platforms specifically addressed to the production of high quality protein drugs can be found elsewhere [15, 16, 21]. The recent development of an endotoxin-free strain of *E. coli* [30] and its application to the fabrication of proteins and protein materials [30–32] paves the road for a cost-efficient and versatile production of proteins intended for biomedical uses by skipping endotoxin removal steps, thus gaining in biosafety and reducing production costs [33]. Hopefully, all these new systems would soon offer improved products in still simple and fully controlled biofabrication approaches.

### Trends in protein biopharmaceuticals

**Fig. 2:**
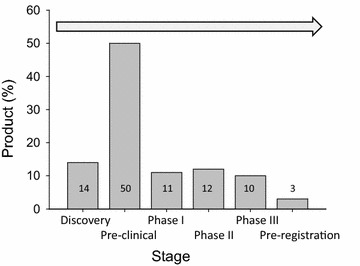
Workflow involved in the development of a new drugs and approximate percentage (*bars* and *numbers*) of recombinant proteins currently in each step [9]

**Fig. 3:**
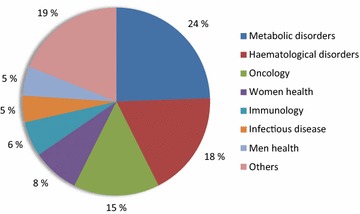
Amount of marketed recombinant proteins (expressed in percentages) applied to each therapeutic area. Coloured in pink, other therapeutic areas (<5 % each) include diseases related to cardiology, central nervous system, ophthalmology and dermatology among others

Nearly 400 recombinant protein-based products have been successfully produced and are approved as biopharmaceuticals [9], a term that refers to therapeutic products generated by technologies that involve living organisms [34]. Other 1300 protein candidates are under development, of which around 50 % are in pre-clinical studies and other 33 % in clinical trials [9] (Fig. 2). In this context, an increase in the number of approvals in next years is predictable. Developed by Eli Lilly & Co in the 70’s, Humulin, a recombinant human insulin fabricated in the bacterium *E. coli* [35], was the first approved biopharmaceutical (by the FDA) in 1982 [36, 37]. Other natural proteins such as hormones, cytokines and antibodies (Orthoclone OKT3) were among the single nine products approved in 1980s (Table 1). Nowadays, the therapeutic areas that have benefited more from recombinant biopharmaceuticals are metabolic disorders (e.g. diabetes type 1, type 2, obesity or hypoglycaemia), haematological disorders (e.g. renal anaemia, haemophilia A, bleeding or clotting disorders) and oncology (e.g. melanoma, breast or colorectal cancer), with 24, 18 and 15 % of the approvals respectively (Fig. 3). In this regard, oncology is a clearly expanding market. In the period 2010–2014, 9 out of 54 approved biopharmaceuticals were antitumoral drugs, cancer representing the most common indication within this period. Digging into the molecular bases of biopharmaceuticals, there is a clear trend towards antibody-based products. Over the same period (2010–2014), 17 of the 54 protein drugs approved were monoclonal antibodies (31.5 %), compared with 11 % over 1980–1989 [22]. Furthermore, among the top ten selling protein biopharmaceuticals globally in 2014 (Table 2), six are antibodies or antibody-derived proteins (Humira, Remicade, Rituxan, Enbrel, Avastin, Herceptin; http://qz.com/349929/best-selling-drugs-in-the-world/).

**Table 1:** Recombinant biopharmaceuticals approved in the 1980s

Product	Cell factory	Therapeutic indication	Year
Humulin	*E. coli*	Diabetes	1982
Protropin	*E. coli*	hGH deficiency	1985
Roferon A	*E. coli*	Hairy cell leukaemia	1986
IntronA	*E. coli*	Cancer, genital warts and hepatitis	1986
Recombivax	*S. cerevisiae*	Hepatitis B	1986
Orthoclone OKT3	Hybridoma cell line	Reversal of acute kidney and transplant rejection	1986
Humatrope	*E. coli*	hGH deficiency	1987
Activase	CHO	Acute myocardial infarction	1987
Epogen	CHO	Anaemia	1989

**Table 2:** Top ten selling protein biopharmaceuticals in 2014

Drug^a^	Active ingredient	Molecule	Sales in billions	Origin
Humira	Adalimumab	Recombinant human monoclonal antibody	12.54	CHO
Sovaldi	Sofosbuvir	Nucleotide analogue polymerase (NS5B) inhibitor	10.28	Chemical
Remicade	Infliximab	Recombinant chimeric, humanized tumor necrosis factor alpha (TNF) monoclonal antibody	9.24	Hybridoma cell line
Rituxan	Rituximab	Recombinant humanized monoclonal antibody	8.68	CHO
Enbrel	Etanercept	Recombinant soluble dimeric fusion protein	8.54	CHO
Lantus	Insulin glargine	Insulin receptor agonist	7.28	*E. coli*
Avastin	Bevacizumab	Recombinant humanized antibody	6.96	CHO
Herceptin	Trastuzumab	Recombinant humanized monoclonal antibody	6.79	CHO
Advair	Fluticasone propionate and salmeterol xinafoate	Glucocorticoid receptor agonist and β-2 adrenergic receptor agonist	6.43	Chemical
Crestor	Rosuvastatin calcium	Antihyperlipedemic agent	5.87	Synthetic

^a^Data according to www.medtrack.com, November 2015

Formerly, biopharmaceuticals were recombinant versions of natural proteins, with the same amino acid sequence as the respective native versions (with only minor modifications, often resulting from the cloning strategy). Since 1990s, a meaningful proportion of the approvals are based on highly modified forms of recombinant proteins. This novel alternative, based on protein or domain fusion and on truncated versions, offers a wide spectrum of possible combinations to obtain novel biopharmaceuticals with different joined activities that are not found together in nature.

### Protein drugs for cancer treatment

Oncology is one of the therapeutic indications that dominate the biopharmaceutical market, as cancer is a major cause of morbidity and mortality worldwide. Surgery and radiotherapy are effective in curing cancer at early disease stages; however, they cannot eradicate metastatic disease. The presence of micrometastases or clinically evident metastases at diagnosis requires their use in combination with genotoxic chemotherapy to increase cure rates [38]. Nevertheless, the success of chemotherapy has been hampered because of its lack of selectivity and specificity, so that the toxicity to normal tissues limits the dose that could be administered to patients. The development of biopharmaceuticals capable of inhibiting specific molecular targets driving cancer (for instance, monoclonal antibodies anti-Her2—Trastuzumab- or anti-VEGF—Bevacizumab-) goes in this direction [39].

**Fig. 4:**
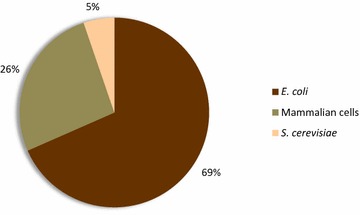
Cell factories used for the production of recombinant biopharmaceuticals against cancer (expressed in percentages)

Among marketed protein biopharmaceuticals, almost 24 % (94 products) are used in antitumoral therapies. Most of these products are used for supportive purposes intended to minimize the side effects of chemotherapy, usually neutropenia or anaemia (some representative examples are shown in Table 3). Nineteen out of those 94 products are true antitumoral drugs, 69 % of which are produced in *E. coli* (Fig. 4) and are based on engineered amino acidic sequences, protein fusions and single protein domains (Table 4).

**Table 3:** Representative examples of supportive protein drugs in cancer

Drug name	Cell factory	Biological role	Mechanism of action	Indications
Filgrastim (Scimax)	*E. coli*	Cytokine	Stimulates hematopoiesis	Bone marrow transplantation and cancer chemotherapy induced neutropenia
Pegfilgrastim (Neupeg)	*E. coli*	Cytokine	Stimulates differentiation, proliferation and activation of the neutrophilic granulocytes	Cancer chemotherapy induced neutropenia
Darbepoetin alfa (Aranesp)	CHO cells	Hormone	Stimulates processes of erythropoiesis or red blood cell production	Anemia associated with chronic renal failure, cancer chemotherapy or heart failure. Myelodysplastic syndrome
Lenograstim (CERBIOS)	CHO cells	Cytokine	Stimulates differentiation, proliferation and activation of neutrophilic granulocytes	Neutropenia associated with cytotoxic therapy or bone marrow transplantation
Epoetin alfa (Binocrit)	CHO cells	Hormone	Stimulates production of oxygen carrying red blood cells from the bone marrow	Anemia associated with chronic renal failure and cancer chemotherapy induced anemia

**Table 4:** Anticancer recombinant biopharmaceuticals approved until March 2015

Drug name	Cell factory	Source	Biological role	Indications
Denileukin diftitox	*E. coli*	Fusion protein	Diphtheria toxin fused to cytokine	Cutaneous T-cell lymphoma
Endostatin	*E. coli*	Modified	Collagen derivative	Non-small cell lung cancer, metastatic colorectal cancer
Aldesleukin	*E. coli*	Modified	Cytokine	Metastatic renal cell carcinoma, metastatic melanoma, kidney cancer, angiosarcoma
Interleukin-2	*E. coli*	Modified	Cytokine	Metastatic melanoma, metastatic renal cell carcinoma
Filgrastim	*E. coli*	Modified	Cytokine	Acute lymphocytic leukaemia, solid tumour
Interferon alpha-2a	*E. coli*	Modified	Cytokine	AIDS-related Kaposi’s sarcoma, follicular lymphoma, cutaneous T-cell lymphoma, melanoma, chronic myelocytic leukaemia, hairy cell leukaemia, renal cell carcinoma, kidney cancer
Interferon alpha-2b	*E. coli*	Modified	Cytokine	AIDS-related Kaposi’s sarcoma, pancreatic endocrine tumour, melanoma, non-Hodgkin lymphoma, leukaemia, hairy cell leukaemia, renal cell carcinoma, multiple myeloma, CML, follicular lymphoma, melanoma
Interferon alpha-1b	*E. coli*	Modified	Cytokine	Renal cell carcinoma, hairy cell leukaemia
Interferon gamma-1a	*E. coli*	Modified	Cytokine	Kidney cancer, sezary syndrome, mycosis fungoides
Tasonermin	*E. coli*	Natural	Cytokine	Soft tissue sarcoma
Molgramostim	*E. coli*	Modified	Growth factor	Myelodysplastic syndrome
Nartograstim	*E. coli*	Modified	Growth factor	Solid tumour
Palifermin	*E. coli*	Fraction	Growth factor	Metastatic renal cell carcinoma, metastatic melanoma
Sargramostim	*S. cerevisiae*	Modified	Growth factor	Acute myelocytic leukaemia
Ziv-aflibercept	CHO cells	Fusion protein	Growth factor receptor fused to IgG1	Metastatic colorectal cancer
Thyrotropin alpha	CHO cells	Modified	Hormone	Thyroid cancer
Trastuzumab biosimilar	CHO cells	Modified	Monoclonal antibody	Breast cancer, gastric cancer, metastatic breast cancer
Rituximab biosimilar	CHO cells	Modified	Monoclonal antibody	Non-Hodgkin lymphoma, chronic lymphocytic leukaemia
Interferon alpha	Human lymphoblastoid cells	Modified	Cytokine	AIDS-related Kaposi’s sarcoma, multiple myeloma, non-Hodgkin lymphoma, CML, hairy cell leukaemia, renal cell carcinoma

**Fig. 5:**
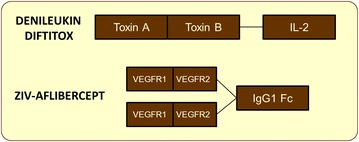
Schematic molecular structure of two marketed recombinant biopharmaceuticals

Clearly, modified protein versions are the most abundant in cancer therapies over natural polypeptides. As relevant examples, Ziv aflibercept is a recombinant fusion protein produced in CHO cells used against colorectal cancer. It consists of portions of each Vascular Endothelial Growth Factor Receptors (VEGFR1 and VEGFR2) fused to the constant fraction (Fc) of a human IgG1 immunoglobulin (Fig. 5). This construct acts as a decoy by binding to VEGF-A, VEGF-B and placental growth factor (PlGF), which activate VEGFR. This trap hinders the interaction between the growth factors and the receptors, inhibiting the VEGF pathway which is involved in the angiogenic process [40]. Denileukin diftitox is a recombinant protein composed of two diphtheria toxin fragments (A and B) and a human interleukin-2 (Fig. 5). Diphteria toxin is a potent exotoxin secreted by *Corynebacterium diphteriae*. Due to its peculiar structure, the whole complex, produced in *E. coli*, is capable of delivering a cytotoxic agent directly to a specific target. There are two main active blocks whose function is firstly to selectively deliver the biopharmaceutical (IL-2) and secondly cause cytotoxicity (toxin A and B) [41]. The fusion protein binds to the IL-2 receptor, which is expressed in cancerous cells (cutaneous T cell lymphoma). Once the toxin moiety is internalized, the catalytic domain promotes cell death through protein synthesis inhibition [42].

As targeted drug delivery for cancer is a most recent and expanding area of research, other non-recombinant, protein-based biopharmaceuticals are also heavily represented. Those mainly include antibody-drug conjugates (ADCs) such as Brentuximab vedotin, Trastuzumab emtansine, or nanoparticle-drug conjugates such as nab-paclitaxel [39, 43]. In these cases, the protein counterpart acts as a targeted vehicle for conventional chemical drugs. Again, this approach pursues the selective drug delivery to specific target cells, aimed to increase antitumoral activity while reducing toxicity on normal cells and the associated side effects.

**Fig. 6:**
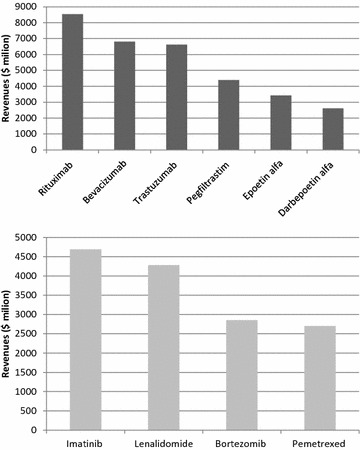
Income provided by recombinant (*top*) and chemical drugs (*bottom*) against cancer in 2013. Figures according to Global Data [9]

Products against cancer that provided the highest revenues in 2013 are represented in Fig. 6. Sixty percent of those products are recombinant proteins, supporting the idea that recombinant protein production is still a rising and promising platform, offering room for important advances in the biopharmaceutical sector.

## Conclusions

In summary, the market and potential for recombinant drugs is expanding by taking advantage of a steady growing spectrum of protein production platforms. Despite the strength of mammalian cell lines as factories, microbial cells and specially *E. coli* are still potent protein factories essentially supported by their versatility and cost-effective cultivation. Recombinant drugs are moving from plain recombinant versions of natural products to more sophisticated protein constructs resulting from a rational design process. Combining protein domains to gain new functionalities is being exploited in drug discovery by exploiting the structural and functional versatility that merge in proteins as extremely versatile macromolecules.

## Abbreviations

AIDS: acquired immune deficiency syndrome
ADCs: antibody-drug conjugates
CHO: chinese hamster ovary
CML: chronic myelogenous leukemia
Fc: constant fraction
FDA: food and drug administration
hGH: human growth hormone
IL: interleukin
PlGF: placental growth factor
VEGF: vascular endothelial growth factor
VEGFR: vascular endothelial growth factor receptor
